# DBP-CanPred: a machine learning model for predicting cancer-causing mutations in DNA-binding proteins

**DOI:** 10.3389/fbinf.2026.1920228

**Published:** 2026-08-28

**Authors:** Amit Phogat, Sowmya Ramaswamy Krishnan, Medha Pandey, M. Michael Gromiha

**Affiliations:** Department of Biotechnology, Bhupat and Jyoti Mehta School of Biosciences, Indian Institute of Technology Madras, Chennai, India

**Keywords:** cancer, DNA-binding proteins, driver mutations, machine learning, mutations

## Abstract

**Introduction:**

The fundamental cellular processes, including transcriptional regulation, chromatin organization, and genome maintenance, are regulated by DNA-binding proteins (DBPs). Mutations in DBPs can alter protein-DNA interactions, leading to tumor development. However, identifying such driver mutations remains a major challenge due to limitations of experimental approaches.

**Methods:**

We have trained a machine learning model, DBP-CanPred, to identify driver mutations in DBPs. We used the sequence-derived evolutionary features, as well as structure-based features such as mutation-perturbed structural descriptors.

**Results:**

We evaluated DBP-CanPred using a curated test set, achieving an AU-ROC of 0.86 and a balanced accuracy of 0.79. Further analysis based on substitution-type showed consistent performance across different categories, especially higher performance on charged residues. In addition, we applied the model on an independent dataset and identified potential driver mutations with high confidence scores.

**Discussion:**

The study contributes to understanding mutation patterns in DNA-binding proteins and supports variant interpretation in cancer research.

## 1 Introduction

Biological processes such as regulation of gene expression, DNA replication, repair, recombination, and chromatin organization depend on specific and non-specific protein-DNA interactions. These interactions are mediated through evolutionarily conserved structural motifs, including helix-turn-helix, homeodomains, leucine zippers, and various zinc-finger domains (Luscombe et al., 2000). DNA-binding proteins (DBPs) recognize target DNA sequences through specific amino acid-base interactions, non-specific protein-DNA contacts, and DNA shape features, which determine the binding affinity and specificity (Gromiha et al., 2004; Rohs et al., 2009). Genetic variations in such DBPs can be classified into either neutral mutations with little functional impact or driver mutations that disrupt the protein structure and function. Mutations at protein-DNA interfaces or within DNA-recognition domains can weaken binding affinity, compromise sequence specificity, or lead to aberrant target recognition through non-specific interactions (Nishi et al., 2013). Integrated analysis of somatic mutations from cancer genome sequencing studies and protein structural information in transcription factors including TP53, RUNX1, TP63, STAT1, and RBPJ have demonstrated that missense mutations in DNA-binding proteins often perturb DNA-binding or functional interfaces (Ashworth et al., 2014).

In TP53, most driver missense mutations cluster within the DNA-binding domain, and recurrent missense substitutions at hotspot residues such as R175, R248, and R273 contribute to the development of multiple cancer types, including breast, connective tissues, brain, and gastrointestinal tumors (Olivier et al., 2010). The recurrent mutations in the forkhead DNA-binding domain of FOXA1 reprogram androgen receptor binding and drive prostate cancer progression, castration resistance, and neuroendocrine differentiation (Dong et al., 2023). Missense mutations such as P286R, S297F, V411L, A456P, and S459F in the exonuclease proofreading domain of POLE are observed across endometrioid ovarian, colorectal, and urothelial cancers, resulting in tumors with very high mutation rates and unique clinical patterns (Kögl et al., 2024). The MYC-MAX complex regulates gene expression by binding E-box DNA elements to control transcriptional amplification, and in multiple myeloma, missense mutations in MAX at key DNA-recognition residues such as R35, R36, and R60 disrupt E-box binding and alter MYC-dependent transcription (Wang et al., 2017). The mutations such as L309P, R339W/Q, R377H, and G420D in zinc-finger domain of CTCF (regulator of transcription and chromatin architecture), disrupt DNA binding leading affecting gene regulation and cellular proliferation (Bailey et al., 2021). In T-cell lymphoma, mutations including D427H, E616G, E616K, and E696K drive constitutive STAT3 activation and enhance DNA binding to target promoters, including PDL1, resulting in immune evasion (Song et al., 2018). The ATPase-domain mutations specifically M781I/R, E821K, T910M, R1135W, and G1232 S/C in SMARCA4/BRG1, a transcription regulator, drive medulloblastoma and oligodendroglioma by impairing chromatin remodeling (Navickas et al., 2023). In summary, mutations in DBPs affect their DNA-binding activity, leading to the development of various types of cancer that affect transcription regulation and chromatin organization. Nevertheless, identifying these driver mutations experimentally is expensive and labor-intensive, underscoring the need for fast and reliable computational approaches (Luo et al., 2023; Lam et al., 2023).

Several computational methods are available for predicting the pathogenicity of missense mutations, including rule-based methods, machine learning, and deep learning models. These methods assess mutation effects using features such as evolutionary information, structural context, and amino acid properties. Meta-predictors further enhance prediction accuracy by combining outputs from multiple individual tools (Suybeng et al., 2020). Rule-based approaches include tools such as SIFT4G (Vaser et al., 2016), MutationAssessor (Reva et al., 2011), PROVEAN (Choi et al., 2012) and LIST-S2 (Malhis et al., 2020). Machine learning-based predictors like MutationTaster (Schwarz et al., 2010), PolyPhen-2 (Adzhubei et al., 2013), MetaSVM (Kim et al., 2017), DEOGEN2 (Raimondi et al., 2017), M-CAP (Jagadeesh et al., 2016), FATHMM-XF (Rog et al., 2018), ClinPred (Alirezaie et al., 2018), CADD (Rentzsch et al., 2019), MutScore (Quinodoz et al., 2022), and PHACTboost (Dereli et al., 2024). Deep learning-based frameworks include DANN (Quang et al., 2015), PrimateAI (Sundaram et al., 2018), MutPred (Pejaver et al., 2020), MVP (Pejaver et al., 2020), AlphaMissense (Cheng et al., 2023), ESM1b (Brandes et al., 2023), and MutFormer (Jiang et al., 2023). Existing variant effect prediction methods show substantial heterogeneity in performance across genes and protein families, reflecting the influence of protein-level factors such as biological function, structural properties, evolutionary constraint, and intrinsic disorder, which are not uniformly captured by current models (Fawzy and Marsh, 2024). Previous studies have shown that relying strictly on consensus among multiple variant effect predictors can discard potentially informative variants when predictions disagree. Consequently, context-specific predictors have been shown to outperform generic and consensus-based methods (de la Campa et al., 2017). Therefore, TMSNP was developed for predicting mutation effects in membrane proteins (Garcia-Recio et al., 2021), machine learning-based models have been proposed to predict gain- and loss-of-function effects in voltage-gated ion channels (Heyne et al., 2020), ZFP-CanPred was introduced for cancer driver mutation prediction in zinc-finger proteins (Phogat et al., 2025), and deep learning approaches have been applied to assess mutation-induced structural changes in intrinsically disordered proteins (Seth and Bhattacharya, 2025). However, DNA-binding proteins have not been addressed specifically by existing prediction approaches. Moreover, DNA-binding proteins are underrepresented in the datasets commonly used for model training, which can reduce predictive accuracy for this protein class. Notably, prior studies have shown that incorporating DNA-binding proteins into domain-adapted training can improve the performance of general models such as ESM (Zeng et al., 2024). These observations suggest that existing methods may not capture mutation effects specific to DNA-binding proteins, highlighting the need for DBP-specific prediction models.

In this work, we have trained a machine learning model, DBP-CanPred, for identifying driver missense mutations in DBPs by leveraging (i) mutations observed in cancer samples and (ii) AlphaFold-predicted structures, reducing bias caused by curated human annotations, and the model learns from both structural and sequence-derived features. DBP-CanPred demonstrated improved discriminative performance on the test set, with an AU-ROC of 0.86 and balanced accuracy of 0.79. We used our model to identify driver mutations from a dataset prepared using COSMIC v103 variants. Notably, 80% of mutations received higher scores from the model, suggesting that it captures patterns associated with cancer-causing variants. Accurate prediction of driver missense mutations in DNA-binding proteins by DBP-CanPred can support cancer research and assist clinical interpretation of variants linked to altered protein-DNA interactions.

## 2 Materials and methods

### 2.1 Dataset

**FIGURE 1:**
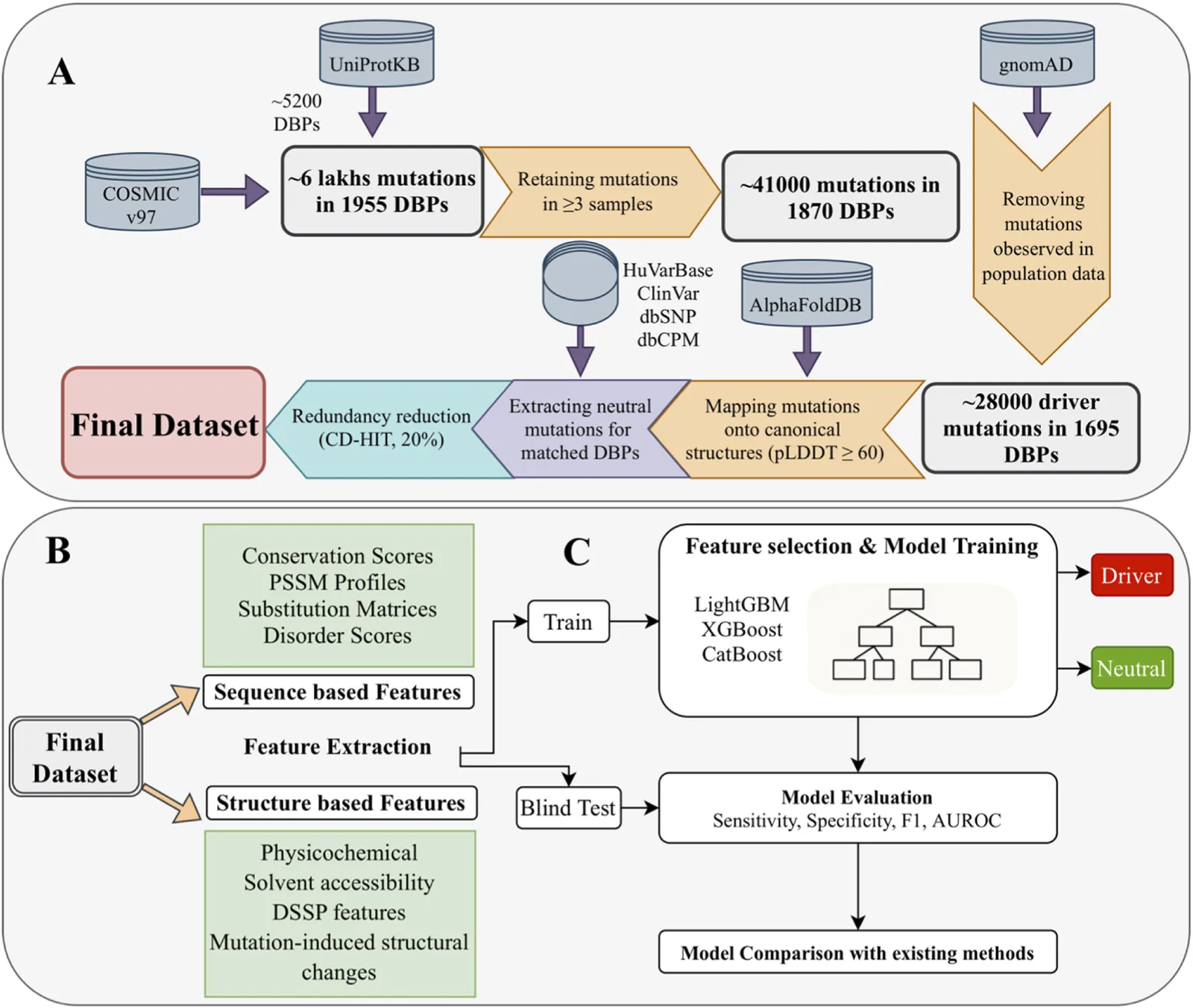
Overview of the DBP-CanPred workflow. **(A)** Shows dataset construction pipeline, missense mutations were obtained from COSMIC and mapped to DNA-binding proteins from UniProtKB. Recurrent mutations (≥3 samples) were retained, germline variants from gnomAD were removed, and neutral mutations were curated from HuVarBase, ClinVar, dbSNP, and dbCPM. Redundancy was reduced using CD-HIT (20%), and mutations were mapped onto AlphaFold structures (pLDDT ≥60) to obtain the final dataset. **(B)** Feature extraction workflow showing the sequence-based and structure-based features used for model development. **(C)** Model development workflow. Extracted features were subjected to feature selection, model training, and hyperparameter optimization, followed by evaluation on an test set and comparison with existing prediction methods.

An overview of the DBP-CanPred workflow is presented in Figure 1. Cancer-associated missense mutations were obtained from the COSMIC database (v97) (Forbes et al., 2017). We collected DNA-binding proteins from UniProtKB (Boutet et al., 2016) annotations, and COSMIC mutations corresponding to these proteins were curated. For each missense mutation, we calculated recurrence across independent tumor samples, and mutations observed in at least three distinct samples were considered (Phogat et al., 2025; Pandey et al., 2022; Pandey and Gromiha, 2023). We removed background germline variation by retaining only mutations unobserved in gnomAD (Karczewski et al., 2020), as demonstrated in previous studies (Cheng et al., 2023), which are designated as driver mutations. We retrieved canonical tertiary structures from the AlphaFold database (Varadi et al., 2024), as complete experimental structures were not available for all proteins in our dataset. We mapped mutations onto structures with a predicted local distance difference test (pLDDT) score of at least 60, and the resulting set was used to construct the driver mutation dataset. We extracted benign or likely benign mutations, hereafter referred to as neutral mutations, from ClinVar (Landrum et al., 2018), dbSNP (Smigielski et al., 2000), HuVarBase (Ganesan et al., 2019), and dbCPM (Yue et al., 2020) for proteins, which contain driver mutations. Neutral mutations were compared against the driver mutation set, and any overlapping variants were removed to ensure mutual exclusivity between the two datasets. We removed redundancy arising from sequence similarity using CD-HIT (Fu et al., 2012) with a sequence identity threshold of 20%, ensuring stringent filtering of homologous sequences. We also evaluated higher identity thresholds (30%–50%) and observed comparable redundancy reduction, with minimal impact on dataset composition. Therefore, a 20% threshold was selected to maximize diversity while avoiding potential bias from closely related sequences.

We partitioned the curated dataset into training and test sets using a protein-level splitting strategy, i.e., each protein was assigned to a single set, preventing information leakage. The approach resulted in a dataset split of 81% and 19% for the train and test sets, respectively. Furthermore, the test set included a subset of amino acid substitutions absent from the training set, facilitating evaluation of unseen variants.

### 2.2 Feature extraction

We have computed various features using sequence, structure and mutation information.

#### 2.2.1 Sequence-based features

##### 2.2.1.1 Conservation-based features

We computed 18 different residue-level conservation scores using the AACon tool (MacGowan et al., 2020) (Supplementary Table S1). Conservation scores quantify the evolutionary constraint at each residue position, where highly conserved residues typically indicate functional or structural importance. These scores are derived from multiple sequence alignments of homologous protein sequences. We extracted homologous sequences from the UniProt database (Boutet et al., 2016), followed by multiple sequence alignment to generate the required profiles. For each mutation, we used the conservation score corresponding to the mutated residue position as a feature.

##### 2.2.1.2 Position-specific scoring matrices (PSSM) profiles

PSSMs capture evolutionary conservation patterns by representing the substitution probability of each amino acid at every residue position, derived from multiple sequence alignments of homologous sequences. We generated PSSMs for each protein sequence using PSI-BLAST (Bhagwat and Aravind, 2007) by searching against the UniProt database (Boutet et al., 2016). For each mutation, we extracted the 6 different PSSM based features, which reflect the evolutionary preference or tolerance of that mutation (Supplementary Table S2).

##### 2.2.1.3 Intrinsically disorder scores

To measure the propensity of each residue to lack a stable tertiary structure, the intrinsic disorder scores were computed using the IUPred2A tool (Mészáros et al., 2018) from protein sequences. We derived 4 disorder-based features, including short- and long-disorder propensities, a binary feature for disorder state, and a local window-based feature calculated over an 11-residue window (Supplementary Table S3). These features quantify the effect of mutations on structural flexibility.

##### 2.2.1.4 Substitution matrix scores

Substitution matrix scores quantify the likelihood of amino acid replacements based on observed substitution patterns in evolutionarily related protein sequences. We extracted 24 substitution scores for each mutation using a diverse set of amino acid substitution matrices (Nikam and Gromiha, 2019) (Supplementary Table S4), including widely used BLOSUM matrices (BLOSUM45) as well as context-specific and structure-informed substitution matrices.

#### 2.2.2 Structure-based features

The descriptions of structure-based features are discussed below and provided in Supplementary Table S5.

##### 2.2.2.1 Physicochemical properties

A total of 49 amino acid physicochemical properties representing physical, chemical, conformational, and energetic characteristics were collected (Gromiha et al., 1999). These properties have been widely used to describe protein structure and function and to assess the effects of amino acid substitutions (Pandey et al., 2022; Pandey and Gromiha, 2023). We quantified the local physicochemical environment of each mutation by considering neighboring residues within a 5 Å all-atom distance cutoff (Salamanca Viloria et al., 2017; Ramaraj et al., 2012) around the mutation site. For each mutation, we computed features by comparing the physicochemical property of the mutant residue with the distribution of the same property among its structural contacts.

Specifically, for each physicochemical property p, we calculated the mean (Equation 1) and standard deviation of property values across contact residues (Equation 2), and standardized the mutant residue value using a z-score formulation (Equation 3). The resulting value reflects the deviation of the mutant residue from its local physicochemical environment.

$$\mu_{p}=\frac{1}{N_{i}}\sum_{i=1}^{N}x_{i}^{\left(p\right)}$$ (1)

$$\sigma_{p}=\sqrt{\frac{1}{N}\sum_{i=1}^{N}{\left(x_{i}^{\left(p\right)}-\mu_{p}\right)}^{2}}$$ (2)

$$z_{p}=\frac{\left(x_{mut}^{\left(p\right)}-\mu_{p}\right)}{\sigma_{p}}$$ (3)

where $x_{i}^{\left(p\right)}$ ​represents the value of property *p* for the *i*th neighbouring residue, $x_{mut}^{\left(p\right)}$ denotes the corresponding value for the mutant residue, and N is the number of contacts. In cases where, $\sigma_{p}=0$ , the z-score was set to zero. These features capture the compatibility of the mutant residue with its local physicochemical environment.

##### 2.2.2.2 Solvent accessibility

We computed mutated-site solvent accessibility using the *FreeSASA* (Mitternacht, 2016) tool, based on protein tertiary structures. We extracted four relative accessibility features (rSASA) corresponding to polar, apolar, main-chain, and side-chain components of a given residue. The features quantify residue solvent exposure, indicating its structural positioning.

##### 2.2.2.3 Secondary structure-based features

We computed secondary structure-based features from protein structures using the DSSP algorithm (Hekkelman et al., 2025). To capture the local structural configuration, we calculated 10 features at the mutated residue position, including secondary structure, dihedral angles, and hydrogen bond descriptors.

##### 2.2.2.4 Mutation-induced structural perturbations features

We computed a set of features, mutation-induced structural perturbations, quantifying the physicochemical and structural changes introduced by amino acid substitutions. The features calculate the shift in hydrophobicity, charge, and residue volume, weighted by side-chain relative solvent accessibility. Specifically, features were calculated as Equations 4–6:

$$\Delta H_{shift}=\left(H_{mut}-H_{wt}\right)\times {RSA}_{side\;chain}$$ (4)

$$\Delta C_{shift}=\left(C_{mut}-C_{wt}\right)\times {RSA}_{side\;chain}$$ (5)

$$\Delta V_{shift}=\left(V_{mut}-V_{wt}\right)\times \left({1-RSA}_{side\;chain}\right)$$ (6)

where *H*, *C*, and *V* denote hydrophobicity, charge, and volume, respectively. Hydrophobicity values were obtained from a previously reported hydrophobicity scale (Gromiha et al., 1999; Akiba et al., 2019), and residue charges were assigned based on amino acid properties as an approximation to capture general physicochemical trends. In contrast, the relative number of atoms per amino acid was used as an approximate measure of residue volume. Additionally, we derived features using secondary structure information to encode helix-specific, sheet-specific, and loop-associated changes. Further, we quantified interactions, including hydrophobic, salt bridges, aromatic, and hydrogen bonds, between wild-type and mutant residues based on the contacting residues. For each interaction type *t*, the change was defined as Equation 7:

$$\Delta I_{t}=I_{t}^{mut}-I_{t}^{wt}$$ (7)

where $I_{t}^{wt}$ and $I_{t}^{mut}$ denote the number of interactions of type *t* formed by the wild-type and mutant residues, respectively. The types of interactions were characterized according to residue property groupings, comprising hydrophobic, charged, polar, and aromatic classes. Also, a binary feature for proline involvement was included to account for its unique structural constraints. Altogether, these features account for the gain or loss of interaction potential induced by mutations within the local structural context.

### 2.3 Model development

#### 2.3.1 Feature selection and model development

We extracted 127 features using protein sequence and structural information. We selected CatBoost, a machine learning algorithm, as the primary classifier due to its consistent performance during our preliminary experiments as compared to other algorithms. We performed forward feature selection with stratified 5-fold cross-validation on the training set, adding features based on the area under the receiver operating characteristic curve (AU-ROC). After feature selection, we optimized the model’s hyperparameters using Optuna (Akiba et al., 2019) within a cross-validation framework, and the final model was selected based on cross-validation AU-ROC.

#### 2.3.2 Model performance evaluation

To evaluate our model’s prediction performance for identifying driver mutations from neutral mutations, we employed a set of matrices defined as follows (Equations 8–12):

$$Accuracy=\left(TP+TN\right)/\left(TP+FN+FP+TN\right)$$ (8)

$$\mathrm{Sensitivity}=TP/\left(TP+FN\right)$$ (9)

$$Specificity=TN/\left(TN+FP\right)$$ (10)

$$Balanced\;accuracy=\left(Sensitivity+Specificity\right)/2$$ (11)

$$F1-score=TP/\left(TP+\frac{\left(FP+FN\right)}{2}\right)$$ (12)

where TP, TN, FP, and FN denote true positives, true negatives, false positives, and false negatives, respectively. We considered driver mutations as the positive class, and neutral mutations as the negative class. In addition to these metrics, model performance was assessed using AU-ROC.

## 3 Results

### 3.1 Dataset curation and mutation distribution

We collected 624,442 missense mutations mapped to 1,955 DBPs from COSMIC v97 (Forbes et al., 2017). We filtered mutations based on recurrence across independent tumor samples (≥3 occurrences), reducing the dataset to 41,436 mutations corresponding to 1,870 DBPs. We further removed background germline variation using gnomAD, resulting in 28,839 driver mutations across 1,695 proteins.

We mapped these mutations to canonical tertiary structures from the AlphaFold database (Varadi et al., 2024), thereby removing redundancy arising from isoform-specific mappings. This resulted in structural coverage for 9,807 driver and 7,167 neutral mutations across 1,589 proteins. This structurally mapped dataset was used for downstream structure-based analysis. For model development and structural contact-based analysis, the dataset was further refined by applying a structural confidence threshold (pLDDT ≥60), resulting in 6,246 driver and 3,908 neutral mutations across 1,042 proteins. Finally, we removed redundancy using CD-HIT at a 20% sequence identity threshold, resulting in a non-redundant dataset comprising 7,140 mutations across 578 proteins, including 4,095 driver and 3,045 neutral mutations. Using a strict protein-level splitting strategy, we partitioned the final dataset into training and test sets, comprising 3578 driver and 2229 neutral mutations in the training set, and 517 driver and 816 neutral mutations in the test set, ensuring no overlap of proteins between the splits.

To analyze mutation distribution at protein-DNA interfaces, we obtained experimentally resolved protein-DNA complex structures from the PDB (Berman et al., 2000) for 207 proteins. To quantify the relative enrichment of driver mutations, the odds ratio was calculated (Equation 13).

$$Odds\;ratio=\frac{N_{d}/N_{d}^{total}}{N_{n}/N_{n}^{total}}$$ (13)

where, $N_{d}$ , and $N_{n}$ denote the number of driver and neutral mutations within the specified region, respectively, where $N_{d}^{total}$ and $N_{n}^{total}$ are total number of driver and neutral mutations, respectively.

**FIGURE 2:**
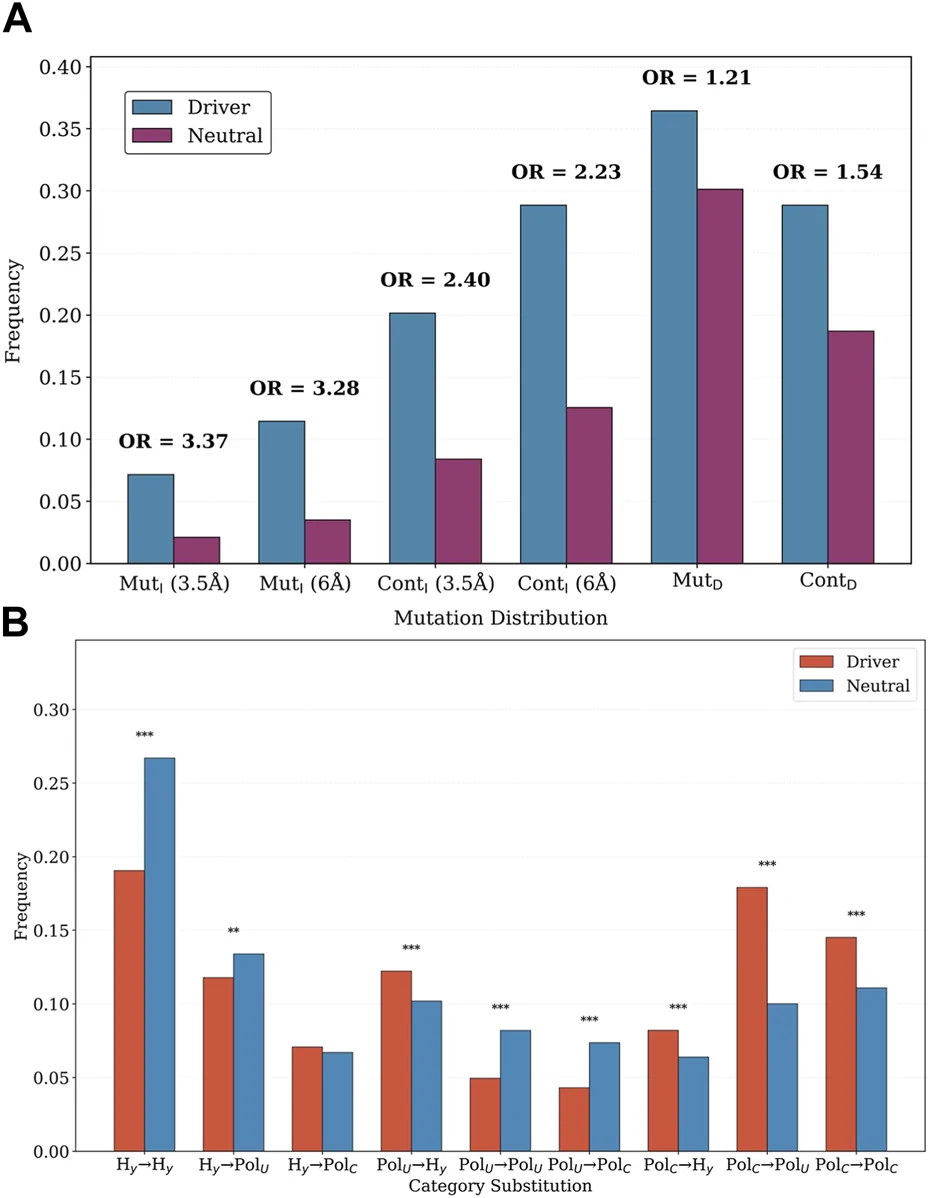
**(A)** Enrichment of driver and neutral mutated sites at protein-DNA interfaces, contacts of mutated sites and DNA-binding domains. **(B)** Comparison of amino acid substitution category frequencies between driver and neutral mutations. Statistical significance is indicated (*P < 0.05, **P < 0.01, ***P < 0.001). Mut: mutation; I: interface, Cont: contacts; D: domain; H_y_: Hydrophobic, Pol_C_:Polar Charged; Pol_U_: Polar Uncharged.

The Odds ratio obtained at different distance cutoffs and types of amino acids are presented in Figure 2A; Supplementary Table S6. We identified DNA-binding interface residues using distance cutoffs of 3.5 Å and 6 Å between protein and DNA atoms, capturing direct and proximal interaction regions, respectively. We observed a clear enrichment of driver mutations at protein-DNA interfaces compared to neutral mutations, with odds ratios of 3.37 and 3.28 at 3.5 Å and 6 Å distance cutoffs, respectively.

To assess indirect structural associations, we analyzed the local structural contacts of mutated residues using the high-confidence structural dataset (pLDDT ≥60), where contacts were defined using a 5 Å all-atom distance cutoff. We observed that mutations associated with structural contacts overlapping DNA-binding interface residues showed higher enrichment in driver mutations compared to neutral mutations, with odds ratios of 2.40 and 2.23 at 3.5 Å and 6 Å interface distance cutoffs, respectively.

We also examined whether a similar pattern exists within DNA-binding domains. Domain annotations were obtained from InterPro (Paysan-Lafosse et al., 2023) for 985 proteins, enabling assessment of mutation distribution within domain regions. We observed that driver mutations were more frequently localized within DNA-binding domains compared to neutral mutations, with an odds ratio of 1.21, suggesting modest enrichment within functional domain regions. Similarly, using the high-confidence structural dataset, we analyzed whether structural contacts of mutated residues overlapped DNA-binding domains. Driver mutations showed a higher association with DNA-binding domain-overlapping structural contacts compared to neutral mutations, with an odds ratio of 1.54. These findings indicate that driver mutations preferentially localize not only at protein-DNA interfaces and within domain regions, but also in their proximal structural neighborhoods, suggesting that they may influence DNA-binding either directly or indirectly by perturbing local structural and functional contexts, whereas neutral mutations are less associated with such critical regions, as reported in earlier studies (Engin et al., 2016; Porta-Pardo et al., 2015; Porta-Pardo and Godzik, 2014).

In addition, we analyzed different amino acid substitution patterns between driver and neutral mutations using the structurally mapped dataset (Figure 2B). We observed that neutral mutations were frequently associated with hydrophobic-to-hydrophobic (H_y_→H_y_), and polar-uncharged substitutions (H_y_→Pol_C_, Pol_U_→Pol_C_, and Pol_U_→Pol_U_). In comparison, driver mutations were predominantly enriched in polar charged residues, notably Pol_C_→Pol_C_ and Pol_C_→Pol_U_ substitutions. The observations are consistent with previous analysis of amino acid substitution tendencies among cancer associated mutations (Anoosha et al., 2016). We further examined the local structural connectivity by analysing the number of contacts of mutated site (Supplementary Figure S1). The analysis showed that driver mutations are associated with a greater number of contacts than neutral mutations, suggesting a denser local structural environment. These results suggest that driver mutations are more likely to occur in structurally constrained and interaction-rich regions, whereas neutral mutations tend to preserve physicochemical properties and are associated with less densely connected environments, consistent with existing studies (Chillón-Pino et al., 2024).

### 3.2 Model performance

**FIGURE 3:**
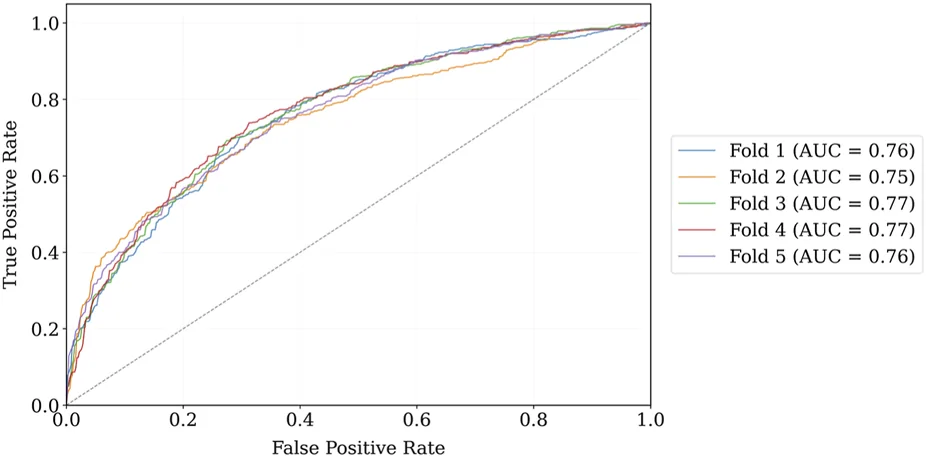
ROC curves from five-fold cross-validation of the selected DBP-CanPred model.

We performed forward feature selection on the training dataset using stratified five-fold cross-validation, yielding an optimal subset of 31 features (Supplementary Table S7). We trained the CatBoost model using the selected 31 features, followed by hyperparameter optimization (Supplementary Table S8). The final model, DBP-CanPred, achieved a mean cross-validation AU-ROC of 0.76 (Figure 3; Supplementary Table S9). For comparison, the performance of LightGBM and XGBoost models trained using the same feature set is provided in Supplementary Table S10. To improve prediction reliability, we used a dual-threshold classification approach to separate confident predictions from uncertain cases. We optimized the threshold values using the training dataset by analyzing the distribution of prediction scores and balancing the model’s performance with prediction coverage. Based on the analysis, we classified mutations with probability scores below 0.46 and above 0.60 as confident neutral and driver mutations, respectively. Conversely, intermediate prediction scores were considered uncertain and were excluded from confidence-based performance evaluation. The performance of DBP-CanPred on the confident subsets along with overall AU-ROC of the training and test datasets is summarized in Table 1.

**TABLE 1:** DBP-CanPred performance on confident training and test dataset.

Dataset	Sensitivity	Specificity	Balanced accuracy	F1	AU-ROC	Coverage	AU-ROC Overall*
Train	0.84	0.73	0.78	0.84	0.84	0.75	0.81
Test	0.85	0.73	0.79	0.77	0.86	0.77	0.83

* AU-ROC overall: AU-ROC including uncertain mutations with a coverage of 100%.

The model showed stable performance on both datasets, with balanced accuracies of 0.78 and 0.79 on the training and test sets, respectively. Similarly, AU-ROC values of 0.84 and 0.86 indicated stable discrimination across both datasets. DBP-CanPred maintained a balanced trade-off between sensitivity and specificity, achieving sensitivity values of 0.84–0.85 and a specificity of 0.73. Overall, our model achieved robust, generalizable performance in confident predictions, with consistent predictive ability across the training and test datasets. Subsequent analysis was performed on the confident subsets of the training and test datasets.

### 3.3 Model interpretation and ablation analysis

**FIGURE 4:**
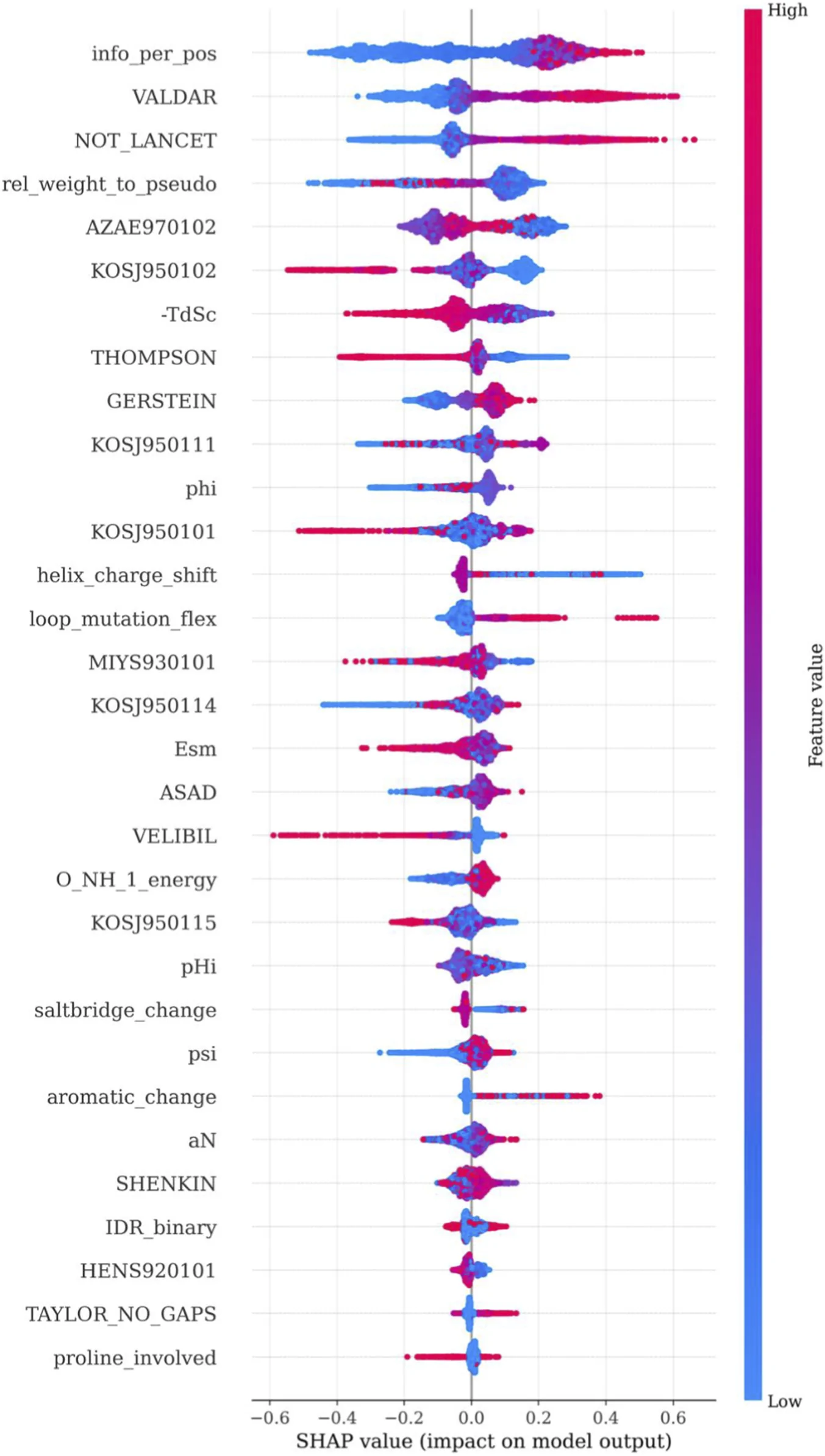
DBP-CanPred model SHAP analysis results showing contributions of each feature to model predictions.

We analyzed feature contributions using SHAP values on the confident training dataset to gain insights into the features contributing in DBP-CanPred predictions (Figure 4). From SHAP analysis, we observed that sequence-derived evolutionary features contributed predominantly, followed by structural features.

Among the sequence-derived features, conservation scores had the greatest influence on the model’s predictions, with higher conservation scores pushing the prediction towards driver mutations. PSSM-derived features were among the top-ranked features, pushing predictions towards driver or neutral depending on the underlying feature value. Substitution matrix features were primarily associated with the prediction of neutral mutations. Compared with other sequence-derived features, disorder-based features influenced model’s predictions minimally. The contribution of evolutionary information and changes in the physicochemical properties of amino acids has been implicated in the study of the functional consequences of mutations (Thusberg et al., 2011; Schaafsma and Vihinen, 2017; Grantham, 1974).

However, all sequence-derived features ranked higher, while structural features contributed complementarily to the model’s predictions. Physicochemical property-derived features contributed strongly towards neutral mutation predictions. While, secondary structure-associated perturbations, such as mutations altering residue charge within helical regions and their impact on loop flexibility, strongly contributed to driver mutation predictions. Structural constraint features from DSSP also provided additional discriminatory power and contributed to neutral mutation predictions. In contrast, the interaction-based features exhibited heterogeneous contributions, with the “*aromatic change*” contribution being positive and the “*proline involved”* values contributing negatively, helping in both driver and neutral mutation prediction. Our analysis suggests that structural context and the impact of mutations on the local structural environment highlight the importance of structural information for mutation impact studies, as reported in previous studies (Babu, 2016; Gerasimavicius et al., 2025).

**FIGURE 5:**
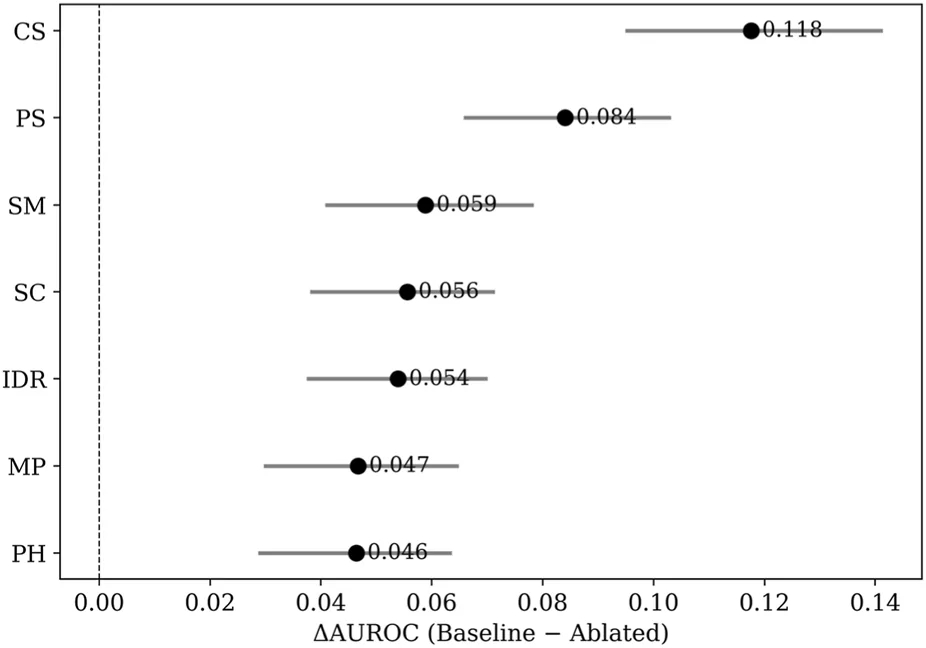
Feature group ablation study illustrating the change in AU-ROC upon removal of each feature group. *SM: substitution matrix features; CS: evolutionary conservation scores; IDR: intrinsically disorder features; PS: PSSM-based features; PH: physicochemical features; SC: structural constraints descriptors; MP: mutation-induced perturbation.

To complement the feature-level interpretation and quantify the relative importance of different feature categories (Supplementary Table S11), we conducted feature-group ablation study using the trained CatBoost classifier to quantify the contribution of different feature categories on test set (Figure 5). A baseline model trained with the complete feature set was first evaluated on the test set. Feature groups were then removed one at a time, and the model was retrained using the remaining features. Model performance after each ablation was assessed on the same test set using AU-ROC. The impact of feature removal was quantified as ΔAU-ROC, defined as the difference between the baseline and the obtained AU-ROC after ablation. Statistical uncertainty was estimated using a paired bootstrap procedure with 1,000 resamples of the test set, and 95% confidence intervals were derived from the empirical ΔAU-ROC distribution. The resulting performance changes revealed distinct differences in the contribution of individual feature groups. The resulting performance changes were broadly consistent with the SHAP-based feature interpretation. Ablation of conservation features led to the largest decrease in performance (ΔAU-ROC ≈0.118), followed by PSSM-derived features (ΔAUROC ≈0.084). Similarly, the drop in AU-ROC from removing substitution-matrix-based features was pronounced, with a significant reduction in performance (ΔAUROC ≈0.059). In contrast, disorder-related (ΔAUROC ≈0.054) and physicochemical features (ΔAUROC ≈0.046) had comparatively smaller effects on model performance. However, secondary structure descriptors (ΔAU-ROC ≈0.056) and mutation-induced perturbation features (ΔAU-ROC ≈0.047) contributed substantially to model performance, indicating the importance of local structural context in mutation classification. Additionally, ablation of all sequence-based and structure-based features reduced the AU-ROC by 0.23 and 0.05, respectively. Also, models trained using sequence or structural features alone (Supplementary Table S12) showed the dominance of sequence-derived features, while structural features provide complementary information to enhance the performance.

Both analyses highlighted the importance of evolutionary, physicochemical, and structural features for model predictions. The findings also demonstrated the significant contribution of mutation-induced perturbation features, emphasizing the impact of mutations on the local structural environment.

### 3.4 Comparison with existing methods

We compared DBP-CanPred against set of widely used variant effect prediction tools on test dataset (Table 2). These include widely used variant effect prediction tools such as SIFT4G (Vaser et al., 2016), PolyPhen-2 (Adzhubei et al., 2013), MetaSVM (Kim et al., 2017), DEOGEN2 (Raimondi et al., 2017), M-CAP (Jagadeesh et al., 2016), FATHMM-XF (Rog et al., 2018), ClinPred (Alirezaie et al., 2018), CADD (Rentzsch et al., 2019), MutScore (Quinodoz et al., 2022), PHACTboost (Dereli et al., 2024), DANN (Quang et al., 2015), PrimateAI (Sundaram et al., 2018), MutPred (Pejaver et al., 2020), MVP (Qi et al., 2021), AlphaMissense (Cheng et al., 2023), ESM1b (Brandes et al., 2023), and MutFormer (Jiang et al., 2023). We collected the precomputed prediction scores from dbNSFP v5 (Liu et al., 2011) for all these methods. A threshold score of 15 was applied to classify variants based on CADD (van der Velde et al., 2017).

**TABLE 2:** Comparison of DBP-CanPred with existing methods on test set.

Tool	Np	Sensitivity	Specificity	BAC	F1	AU-ROC
Generic methods						
Polyphen2	1001	0.69 (0.85)	0.57 (0.73)	0.63 (0.79)	0.60 (0.77)	0.67 (0.86)
MetaSVM	1001	0.47 (0.85)	0.66 (0.73)	0.56 (0.79)	0.48 (0.77)	0.59 (0.86)
M-CAP	973	0.71 (0.85)	0.27 (0.73)	0.49 (0.79)	0.53 (0.77)	0.53 (0.86)
MutPred	845	0.69 (0.85)	0.59 (0.74)	0.64 (0.80)	0.64 (0.79)	0.70 (0.86)
MVP	880	0.24 (0.83)	0.41 (0.74)	0.32 (0.78)	0.22 (0.73)	0.28 (0.86)
PrimateAI	998	0.67 (0.85)	0.88 (0.73)	0.78 (0.79)	0.73 (0.77)	0.85 (0.86)
DEOGEN2	1001	0.43 (0.85)	0.62 (0.73)	0.52 (0.79)	0.45 (0.77)	0.56 (0.86)
ClinPred	1001	0.91 (0.85)	0.50 (0.73)	0.70 (0.79)	0.70 (0.77)	0.79 (0.86)
AlphaMissense	965	0.83 (0.85)	0.74 (0.74)	0.78 (0.80)	0.77 (0.77)	0.84 (0.86)
CADD	1001	0.00 (0.85)	1.00 (0.73)	0.50 (0.79)	0.00 (0.77)	0.75 (0.86)
DANN	1001	1.00 (0.85)	0.06 (0.73)	0.53 (0.79)	0.61 (0.77)	0.63 (0.86)
PHACTboost	1001	0.88 (0.85)	0.61 (0.73)	0.74 (0.79)	0.73 (0.77)	0.84 (0.86)
MutFormer	1001	0.40 (0.85)	0.34 (0.73)	0.37 (0.79)	0.35 (0.77)	0.27 (0.86)
MutScore	999	0.32 (0.85)	0.88 (0.73)	0.60 (0.79)	0.43 (0.77)	0.74 (0.86)
SIFT4G	889	0.68 (0.82)	0.66 (0.73)	0.67 (0.77)	0.60 (0.72)	0.30 (0.86)
ESM1b	1001	0.87 (0.85)	0.59 (0.73)	0.73 (0.79)	0.71 (0.77)	0.23 (0.86)
Cancer-specific methods						
Fathmm-XF	997	0.90 (0.85)	0.53 (0.73)	0.72 (0.79)	0.70 (0.77)	0.78 (0.86)
DBP-CanPred	1023	0.85	0.73	0.79	0.77	0.86

N_p_: predicted mutations; BAC: balanced accuracy; DBP-CanPred performance for corresponding dataset is shown in parentheses.

In comparison, DBP-CanPred outperformed most methods, including SIFT, PolyPhen, MutPred, and MutScore, achieving a balanced accuracy of 0.79. While some methods such as ClinPred, CADD and DANN achieved higher sensitivity or specificity, with reduced performance in other metrics, whereas DBP-CanPred maintained balanced predictive performance. Some methods, including PrimateAI, PHACTboost, and ESM1b, achieved balanced accuracies comparable to DBP-CanPred, but exhibited a greater trade-off between sensitivity and specificity.

Among all existing methods, AlphaMissense gave notable performance; therefore, we performed an additional comparative analysis focusing on uncertain predictions (Table 3). AlphaMissense performance declined on mutations classified as uncertain by DBP-CanPred. Conversely, DBP-CanPred maintained strong predictive performance on uncertain variants identified by AlphaMissense, achieving balanced accuracy of 0.72 and AU-ROC of 0.77. This comparison highlights the improved performance of DBP-CanPred over existing methods, particularly for DNA-binding proteins.

**TABLE 3:** Comparison of AlphaMissense and DBP-CanPred on uncertain variants classified by each model.

Evaluation subset	Sensitivity	Specificity	BAC	F1	AU-ROC
AlphaMissense on DBP-CanPred uncertain variants	0.59	0.72	0.66	0.48	0.71
DBP-CanPred on AlphaMissense uncertain variants	0.79	0.64	0.72	0.62	0.77

### 3.5 Comparison with existing methods based on different substitutions

Furthermore, we compared the methods with DBP-CanPred using balanced accuracies by stratifying mutations based on different substitution categories (Figure 2B) (Supplementary Table S13). The model maintained balanced performance consistently across all substitution categories, particularly mutations involving polar uncharged and polar charged residues. DBP-CanPred outperformed existing methods across multiple categories, achieving the highest balanced accuracies for polar uncharged-to-polar charged (0.80), hydrophobic-to-polar uncharged (0.82), polar charged-to-polar charged (0.84), and polar uncharged-to-hydrophobic (0.83) substitutions. For the other categories, DBP-CanPred maintained robust performance, with balanced accuracies of 0.79 and 0.75 for hydrophobic-to-polar charged and hydrophobic-to-hydrophobic substitutions, respectively. The model performed relatively moderate for polar charged-to-polar uncharged (0.70) and polar charged-to-hydrophobic (0.72) substitution categories. In summary, DBP-CanPred maintained robust and balanced performance across all categories, demonstrating its ability to generalize to diverse amino acid substitutions.

### 3.6 Model performance on frequently mutated genes

We evaluated DBP-CanPred on genes categorized as rarely mutated (1–20 mutations) and frequently mutated (>20 mutations) using the test dataset (Table 4).

**TABLE 4:** DBP-CanPred performance across rarely and frequently mutated genes.

Mutated genes	Drivers	Neutral	Uncertain	Sensitivity	Specificity	BAC
Rare (1–20)	22	23	8	0.74	0.67	0.70
Frequent (>20)	495	793	300	0.85	0.74	0.79

* BAC: balanced accuracy.

The model showed superior performance on frequently mutated genes, achieving a balanced accuracy of 0.79 compared with 0.70 for rarely mutated genes. The performance difference may reflect the greater representation of recurrent mutation patterns and contextual information, likely facilitating more reliable discrimination between driver and neutral mutations. Nevertheless, the model maintained consistent performance across both groups, indicating its applicability across genes with varying mutation frequencies.

### 3.7 Model performance on frequent and rare mutations

Additionally, we also examined DBP-CanPred predictions on mutations with different recurrence in independent tumor samples (Table 5).

**TABLE 5:** Performance of DBP-CanPred across recurrent driver mutations in the test dataset.

Mutation recurrence	Total drivers	Confident predictions	Uncertain	Correctly predicted	Prediction accuracy
3	229	188	41	153	0.81
4	93	81	12	68	0.84
5	54	48	6	40	0.83
6	37	33	4	28	0.85
7	26	22	4	20	0.91
8	18	16	2	15	0.94
9	10	9	1	9	1.00
≥10	50	47	3	43	0.91

We calculated prediction accuracy for each group, defined as the proportion of correctly predicted driver mutations among confident predictions. The model achieved strong performance across all recurrence groups, with prediction accuracy increasing with higher recurrence. The model’s performance showed a modest increase for mutations observed up to 6 times, ranging from 0.81 to 0.85. In contrast, mutations that occurred more than 6 times showed greater prediction accuracies, reaching up to 0.94. These observations suggest that highly recurrent mutations are more likely to drive cancer, and the higher prediction accuracy of DBP-CanPred suggests that the model effectively captures signals associated with driver mutations.

### 3.8 Impact of structural quality on model performance

**FIGURE 6:**
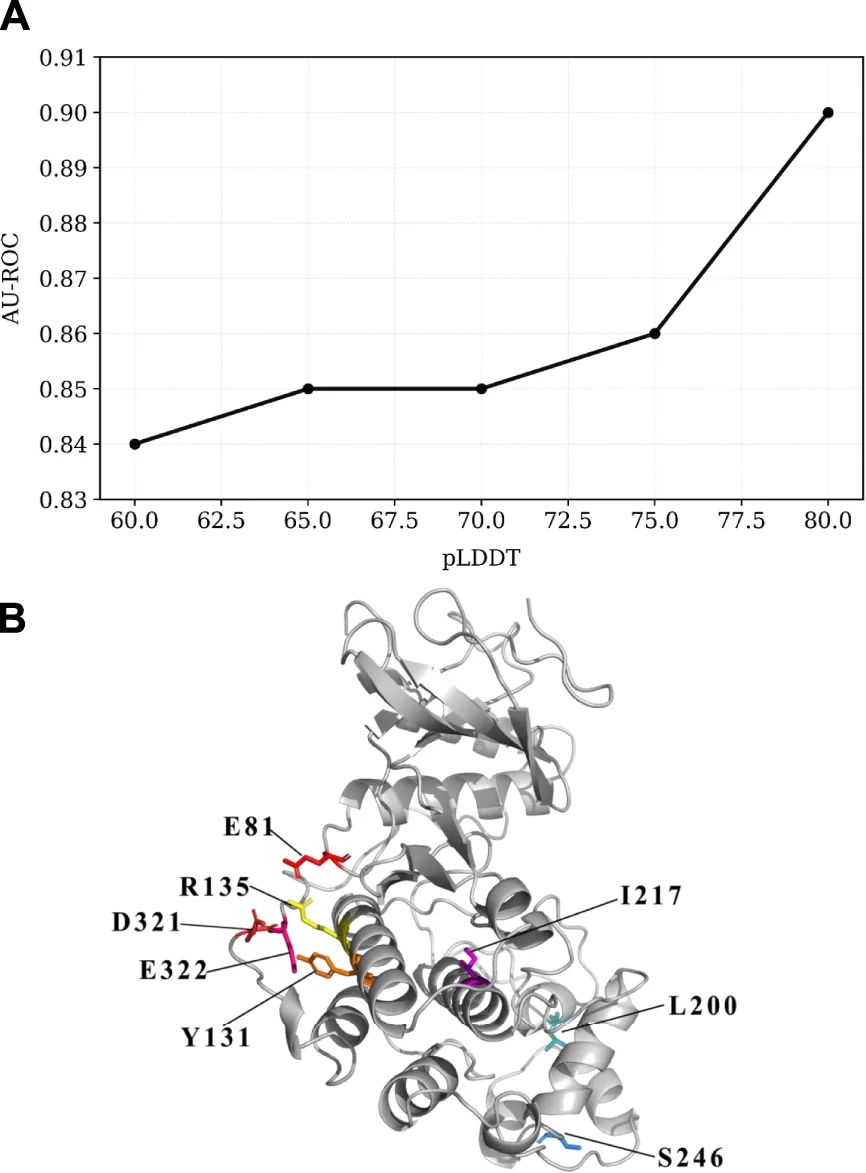
**(A)** Effect of structural confidence (pLDDT) on model performance, showing AU-ROC with increasing pLDDT scores on the training dataset. **(B)** Experimentally resolved structure of MAPK1 (UniProt: P28482, PDB: 4QTE) highlighting mapped mutation sites used for structural validation of DBP-CanPred. Mutated residues are shown as colored stick representations on the monomeric protein structure.

To evaluate the influence of predicted structural quality on model’s predictions, we analyzed DBP-CanPred using training dataset grouped based on pLDDT from AlphaFold structures. We categorized mutations according to increasing pLDDT, and computed corresponding AU-ROC scores for each subset. The results showed a positive association between structural quality and model performance (Figure 6A), suggesting that improved structural quality enhances model predictions. A gradual increase in performance was observed between pLDDT 60 and 75, followed by a more pronounced improvement at higher confidence levels. These results demonstrate that DBP-CanPred achieves more reliable predictions when supported by high-confidence structural models, consistent with existing literature reporting that prediction performance is influenced by the quality of AlphaFold structures (Scardino et al., 2022; Li et al., 2025; Pak et al., 2023). To further validate the influence of structural quality using experimentally resolved structural data (Berman et al., 2000), we selected the protein kinase MAPK1 (UniProt: P28482) from our dataset, for which a complete monomeric experimental structure without missing residues was available. A total of nine mutations from the dataset, comprising eight driver and one neutral mutation, were mapped onto the experimentally resolved structure (Figure 6B).

DBP-CanPred generated confident predictions for eight mutations, with one mutation classified as uncertain. On the confidently predicted subset, the model achieved balanced accuracy of 0.93, with sensitivity of 0.86 and specificity of 1. These results further support the observation that high-quality structural information improves predictive reliability.

### 3.9 Case study

**FIGURE 7:**
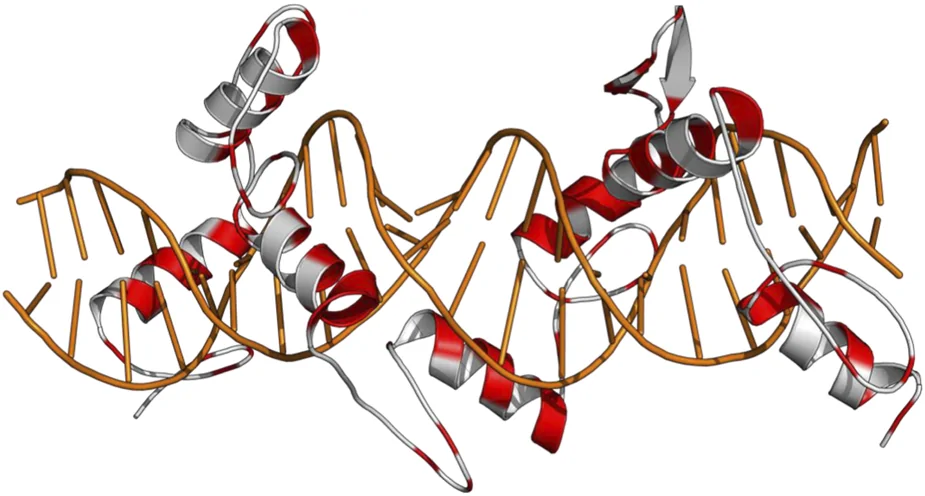
CTCF-DNA complex structure (PDB ID: 8SSS) showing protein-DNA interface residues. DNA is shown in orange, and the 73 identified interface residues are highlighted in red.

To further investigate the DBP-CanPred model, we performed a case study on the DNA-binding protein CTCF using the experimentally resolved protein-DNA complex structure (Berman et al., 2000) (PDB ID: 8SSS) (Figure 7). In cancer, CTCF serves as a context-dependent regulatory factor, where alterations in its function, expression, or dosage contribute to tumorigenesis, genomic instability, and the maintenance of malignant cell survival (Segueni and Noordermeer, 2022). The available structure contains residues 264-461 of the full-length 727-residue protein and encompasses the DNA-binding region involved in direct protein-DNA interactions. Protein-DNA interface residues were identified from the complex structure using the 3.5 Å distance cut-off, yielding a total of 73 interface residues.

To evaluate the mutational landscape of the DNA-binding interface, the DNA molecule was removed and the protein structure was used for feature extraction and prediction. Each of the 73 interface residues were mutated to the remaining 19 amino acids, generating a total of 1,387 possible missense mutations. DBP-CanPred classified 854 mutations as drivers, 133 as neutral, and 400 as uncertain, indicating a strong enrichment of predicted driver mutations within the DNA-binding interface region.

To assess the relevance of these predictions, the generated mutations were compared against variants observed in COSMIC (v102), ClinVar, and gnomAD. Among the COSMIC mutations mapped to interface residues, 40 were observed in a single tumor sample, whereas 14 were recurrently observed in two or more independent tumor samples. ClinVar-associated interface mutations consisted of pathogenic/likely pathogenic alterations and variants of uncertain significance (VUS), whereas gnomAD-associated interface mutations corresponded only to extremely rare population variants with very low allele frequencies (≤10^−7^).

Among the 73 interface residues, 60 sites had prior mutation evidence in at least one database, whereas 13 interface sites had no previously reported mutations. Systematic mutagenesis of the 60 previously observed mutated sites generated 1,140 possible mutations, of which DBP-CanPred classified 713 as drivers, 108 as neutral, and 319 as uncertain. In contrast, mutagenesis of the 13 previously unreported interface sites produced 247 mutations, including 141 predicted drivers, 25 neutral mutations, and 81 uncertain predictions. These findings suggest that several structurally important interface residues lacking prior mutational evidence may still harbor potentially deleterious substitutions. Mutations observed at CTCF interface residues in COSMIC, ClinVar, and gnomAD and their corresponding DBP-CanPred predictions are presented in Supplementary Table S14. Within the COSMIC dataset, DBP-CanPred preferentially classified recurrent mutations as drivers, identifying 10 of 14 recurrent mutations as drivers and none as neutral, whereas 23 of 40 singleton mutations were predicted as drivers. Similarly, out of 24 ClinVar pathogenic interface mutations, 19 were classified as drivers and none as neutral. Further analysis of gnomAD-derived interface mutations showed that 16 of 20 unobserved mutations (allele frequency = 0) were predicted as drivers. In summary, these results highlight that DBP-CanPred prioritizes potential deleterious mutations at DNA-binding interfaces and shows strong concordance with experimentally and clinically observed mutation patterns.

## 4 Application

We applied DBP-CanPred to a curated dataset derived from COSMIC v102 (Zeng et al., 2024), following a similar workflow described in the methodology, considering mutations observed in at least 5 samples. In addition, proteins with ≥40% sequence identity to those in the training dataset were excluded, resulting in a final dataset comprising 471 mutations. The model predicted 130 variants with uncertain classification, while approximately 80% of the confidently classified variants were predicted as drivers by DBP-CanPred.

The model assigned high-confidence scores to a large proportion of mutations, suggesting its ability to identify potentially functional mutations beyond well-characterized cases. The detailed prediction results for this dataset can be downloaded from our web server.

## 5 Web server and data availability

DBP-CanPred is available through a publicly accessible web server that allows users to submit protein mutations to get the predictions. We developed web interface using React with TypeScript and Vite, a single-page application for data submission, job monitoring, and prediction results. We implemented backend in Python using CGI-based scripts to process user inputs, execute feature extraction and the prediction model, and retrieve the results. The server processes each submission through an asynchronous job-based workflow. Each job is assigned a unique identifier, allowing users to monitor its progress and retrieve the prediction results once the analysis is complete.

The web server is hosted on a Linux server maintained by Protein Bioinformatics Laboratory, IIT Madras. The complete training and test datasets used in this study are also provided through the web interface. Details on access, usage, and input formats are provided on the website (https://web.iitm.ac.in/bioinfo3/DBP-CanPred/). The web server will be actively maintained by the authors, with periodic software updates, bug fixes, and compatibility improvements to ensure continued accessibility.

## 6 Discussion

DNA-binding proteins are critical to cellular systems for mediating transcriptional control, chromatin organization, and genome maintenance. Therefore, mutations in these proteins can disrupt protein-DNA interactions, leading to aberrant regulatory outcomes and cancer development. However, DNA-binding proteins remain underrepresented in large-scale mutation datasets used to train generic variant effect predictors, limiting their reliability when applied.

In this study, we analyzed the distribution of driver mutations within functional regions of DNA-binding proteins. These mutations were enriched at protein-DNA interfaces, DNA-binding domains, and in structurally dense regions. Similarly, our analysis of substitution categories showed that driver mutations were enriched in charged residue substitutions. The observations suggest that driver mutations tend to occur at functionally and structurally important sites, thereby directly or indirectly affecting potential interactions.

Building upon these findings, we developed DBP-CanPred to predict driver mutations in DNA-binding proteins by integrating sequence- and structure-based features. Structural descriptors were extracted from predicted AlphaFold structures (pLDDT ≥60). We also examined the feature contribution to the model’s predictions using SHAP values and ablation analysis, revealing that the model’s performance was primarily driven by sequence-based features, especially evolutionary descriptors, whereas structure-derived features further refined predictions.

DBP-CanPred maintained balanced performance across the training and test sets. We compared the model with existing methods, it achieved higher balanced accuracy and AU-ROC on the test set. Unlike several existing methods, DBP-CanPred maintained a balanced trade-off between sensitivity and specificity. We further evaluated DBP-CanPred across specific amino acid substitution categories. The model maintained consistent performance across diverse substitution types, especially mutations involving charged residues and substantial physicochemical changes. In addition, the model demonstrated superior performance on frequently mutated genes and highly recurrent driver mutations, suggesting its ability to identify cancer-driving mutations.

We further examined the impact of predicted structural model quality on the model’s performance. The analysis revealed a positive association between pLDDT scores and AUROC. We validated this observation using an experimentally resolved MAPK1 structure, where DBP-CanPred achieved high balanced accuracy for confidently predicted mutations. These findings highlight the importance of reliable structural information for mutation-effect prediction and demonstrate that AlphaFold-derived structural features can yield biologically meaningful insights.

The CTCF case study further illustrated the utility of DBP-CanPred for systematic exploration of mutational landscapes at protein-DNA interfaces. Comprehensive mutagenesis of interface residues revealed substantial enrichment of predicted driver mutations. Moreover, the strong agreement between DBP-CanPred predictions and mutations reported in existing databases suggests that the model captures functionally important interface perturbations.

Finally, we applied the DBP-CanPred on an independent COSMIC dataset. The enrichment of confident driver predictions suggests that the model captures features associated with cancer-driving mutations. These findings support the use of DBP-CanPred for prioritizing variants that remain functionally uncharacterized.

## 7 Conclusion

In summary, the study presents a systematic analysis of driver mutations in DNA-binding proteins and presents DBP-CanPred, a machine learning-based model for identifying driver mutations. Model’s performance is primarily driven by sequence derived evolutionary features, underscoring the importance of functionally constrained residues in determining mutation impact. Additionally, mutation-induced perturbations in local structural environment further refined DBP-CanPred predictions. The model demonstrates robust performance on the test set, achieving improved balanced accuracy and AU-ROC in comparison with existing predictors while maintaining a balanced sensitivity-specificity trade-off. These results highlight the importance of developing prediction tools for underrepresented protein groups. In this context, accurate prediction of mutation effects in DNA-binding proteins using DBP-CanPred can support cancer research and variant interpretation.

## Data Availability

The DBP-CanPred tool, along with its training dataset, is publicly available at https://web.iitm.ac.in/bioinfo3/DBP-CanPred/.
