# Supplementary material for: DBP-CanPred: a machine learning model for predicting cancer-causing mutations in DNA-binding proteins
Source: Front Bioinform. 2026 Aug 28;6:1920228. doi: 10.3389/fbinf.2026.1920228 (PMC13562013; doi:10.3389/fbinf.2026.1920228)
Supplement: Supplementary file 1 [file Supplementaryfile1.docx]

**DBP-CanPred: A Machine Learning Model for Predicting Cancer-Causing Mutations in DNA-Binding Proteins**

Amit Phogat^1^, Sowmya Ramaswamy Krishnan^1^, Medha Pandey^1^, M. Michael Gromiha^1,#^

^1^Department of Biotechnology, Bhupat and Jyoti Mehta School of Biosciences, Indian Institute of Technology Madras, Chennai 600036, India

^#^Author for correspondence – [gromiha@iitm.ac.in](mailto:gromiha@iitm.ac.in)

**Supplementary information**

**Table S1:** Conservation scores calculated using AACON.

| **Feature** | **Reference** |
| --- | --- |
| KABAT | Wu and Kabat (1970) |
| JORES | Jores et al. (1990) |
| SCHNEIDER | Schneider (1997) |
| SHENKIN | Shenkin et al. (1991) |
| GERSTEIN | Gerstein and Altman (1995) |
| TAYLOR_GAPS | Taylor (1986) |
| TAYLOR_NO_GAPS | Taylor (1986) |
| VELIBIL | Zvelebil et al. (1987) |
| KARLIN | Mirny and Shakhnovich (2001) |
| ARMON | Armon et al. (2001) |
| THOMPSON | Thompson et al. (1997) |
| NOT_LANCET | Pilpel and Lancet (1999) |
| MIRNY | Mirny and Shakhnovich (1999) |
| WILLIAMSON | Williamson (1995) |
| LANDGRAF | Landgraf et al. (1999) |
| SANDER | Sander and Schneider (1991) |
| VALDAR | Valdar and Thornton (2001) |
| SMERFS | Manning et al. (2008) |

**Table S2:** PSSM-based features extracted from PSI-BLAST profiles.

| **Feature** | **Description** |
| --- | --- |
| pssm_mut | Raw PSSM score of the mutant amino acid at the mutation position. |
| pssm_mut_weighted | Weighted observed percentage of the mutant amino acid at the mutation position. |
| pssm_mut_diff | Difference between the mutant and wild-type raw PSSM scores, calculated as (PSSM_mut - PSSM_wt). |
| pssm_weight_diff | Difference between the mutant and wild-type weighted PSSM scores, calculated as (Weighted_mut - Weighted_wt). |
| info_per_pos | Information content at the mutation position reported by PSI-BLAST. |
| rel_weight_to_pseudo | Relative weight of gapless real matches to pseudocounts at the mutation position. |

**Table S3:** Intrinsic disorder features derived from residue-level disorder propensities.

| **Feature** | **Description** |
| --- | --- |
| IDR_short_diff | Difference between the mutant and wild-type short disorder propensities predicted by IUPred2A. |
| IDR_long_diff | Difference between the mutant and wild-type long disorder propensities predicted by IUPred2A. |
| IDR_window_mean_diff | Difference in the mean disorder propensity within an 11-residue window centered at the mutation site between the mutant and wild-type sequences. |
| IDR_binary | Binary indicator denoting whether the mutation changes the disorder state of the residue based on the IUPred2A threshold. |

**Table S4:** Amino-acid substitution matrices

| **ID** | **Description (Reference)** |
| --- | --- |
| AZAE970101 | The single residue substitution matrix from interchanges of spatially neighbouring residues (Azarya-Sprinzak et al., 1997) |
| AZAE970102 | The substitution matrix derived from spatially conserved motifs (Azarya-Sprinzak et al., 1997) |
| HENS920101 | BLOSUM45 substitution matrix (Henikoff-Henikoff, 1992) |
| HENS920102 | BLOSUM62 substitution matrix (Henikoff-Henikoff, 1992) |
| HENS920103 | BLOSUM80 substitution matrix (Henikoff-Henikoff, 1992) |
| GIAG010101 | Residue substitutions matrix from thermo/mesophilic to psychrophilic enzymes (Gianese et al., 2001) |
| HENS920104 | BLOSUM50 substitution matrix (Henikoff-Henikoff, 1992) |
| KOSJ950101 | Context-dependent optimal substitution matrices for exposed helix (Koshi-Goldstein, 1995) |
| KOSJ950102 | Context-dependent optimal substitution matrices for exposed beta (Koshi-Goldstein, 1995) |
| KOSJ950103 | Context-dependent optimal substitution matrices for exposed turn (Koshi-Goldstein, 1995) |
| KOSJ950104 | Context-dependent optimal substitution matrices for exposed coil (Koshi-Goldstein, 1995) |
| KOSJ950105 | Context-dependent optimal substitution matrices for buried helix (Koshi-Goldstein, 1995) |
| KOSJ950106 | Context-dependent optimal substitution matrices for buried beta (Koshi-Goldstein, 1995) |
| KOSJ950107 | Context-dependent optimal substitution matrices for buried turn (Koshi-Goldstein, 1995) |
| KOSJ950108 | Context-dependent optimal substitution matrices for buried coil (Koshi-Goldstein, 1995) |
| KOSJ950109 | Context-dependent optimal substitution matrices for alpha helix (Koshi-Goldstein, 1995) |
| KOSJ950110 | Context-dependent optimal substitution matrices for beta sheet (Koshi-Goldstein, 1995) |
| KOSJ950111 | Context-dependent optimal substitution matrices for turn (Koshi-Goldstein, 1995) |
| KOSJ950112 | Context-dependent optimal substitution matrices for coil (Koshi-Goldstein, 1995) |
| KOSJ950113 | Context-dependent optimal substitution matrices for exposed residues (Koshi-Goldstein, 1995) |
| KOSJ950114 | Context-dependent optimal substitution matrices for buried residues (Koshi-Goldstein, 1995) |
| KOSJ950115 | Context-dependent optimal substitution matrices for all residues (Koshi-Goldstein, 1995) |
| MIYS930101 | Base-substitution-protein-stability matrix (Miyazawa-Jernigan, 1993) |
| MUET010101 | Non-symmetric substitution matrix (SLIM) for detection of homologous transmembrane proteins (Mueller et al., 2001) |

**Table S5:** Structure-based features derived from solvent accessibility, secondary structure annotations, and mutation-induced structural perturbations.

| **Feature** | **Description** |
| --- | --- |
| rel_polar_sasa* | Relative solvent-accessible surface area (SASA) contributed by polar atoms of the mutated residue. |
| rel_apolar_sasa | Relative SASA contributed by apolar atoms of the mutated residue. |
| rel_main_chain_sasa | Relative SASA of backbone atoms at the mutation site. |
| rel_side_chain_sasa | Relative SASA of side-chain atoms at the mutation site. |
| phi | Backbone φ (phi) torsion angle of the mutated residue. |
| psi | Backbone ψ (psi) torsion angle of the mutated residue. |
| NH_O_1_relidx | Sequence-relative index of the primary backbone N-H···O hydrogen-bond acceptor partner. |
| NH_O_1_energy | Energy of the primary backbone N-H···O hydrogen bond. |
| O_NH_1_relidx | Sequence-relative index of the primary backbone O···H-N hydrogen-bond donor partner. |
| O_NH_1_energy | Energy of the primary backbone O···H-N hydrogen bond. |
| NH_O_2_relidx | Sequence-relative index of the secondary backbone N-H···O hydrogen-bond acceptor partner. |
| NH_O_2_energy | Energy of the secondary backbone N-H···O hydrogen bond. |
| O_NH_2_relidx | Sequence-relative index of the secondary backbone O···H-N hydrogen-bond donor partner. |
| O_NH_2_energy | Energy of the secondary backbone O···H-N hydrogen bond. |
| surface_hydro_shift | Change in residue hydrophobicity weighted by side-chain relative solvent accessibility. |
| surface_charge_shift | Change in residue charge weighted by side-chain relative solvent accessibility. |
| core_volume_shift | Change in residue volume weighted by burial |
| helix_charge_shift | Surface charge shift computed only for mutations located in α-helices. |
| sheet_core_stress | Core-weighted volume shift computed only for mutations located in β-sheets. |
| helix_hydro_shift | Surface hydrophobicity shift computed only for mutations located in α-helices. |
| loop_mutation_flex | Flexibility-related perturbation score for mutations occurring in loop or coil regions. |
| hydrophobic_change | Difference in the number of hydrophobic interactions formed by mutant and wild-type residues. |
| saltbridge_change | Difference in the number of salt bridges formed by mutant and wild-type residues. |
| hbond_change | Difference in the number of hydrogen bonds formed by mutant and wild-type residues. |
| aromatic_change | Difference in the number of aromatic interactions formed by mutant and wild-type residues. |
| proline_involved | Binary indicator denoting whether the wild-type or mutant residue is proline. |

**Table S6:** Distribution of driver and neutral mutations and their contacts across DNA-binding interfaces and domains.

| **Analysis** | **Driver** | | | **Neutral** | | | **OR** |
| --- | --- | --- | --- | --- | --- | --- | --- |
|  | **Proteins** | **Sites (Mutations)** | **Sites at interface/domain (Mutations)** | **Proteins** | **Total Sites (Mutations)** | **Sites at interface/domain (Mutations)** |  |
| DNA interface (3.5 Å) | 207 | 1985 (2665) | 142 (209) | 166 | 2264 (2447) | 48 (50) | 3.37 |
| DNA interface (6 Å) | 207 | 1985 (2665) | 227 (347) | 166 | 2264 (2447) | 79 (83) | 3.28 |
| DNA interface contacts (3.5 Å) | 133 | 1260 (1897) | 254 (459) | 104 | 1226 (1296) | 103 (107) | 2.40 |
| DNA interface contacts (6 Å) | 133 | 1260 (1897) | 353 (628) | 104 | 1226 (1296) | 154 (161) | 2.23 |
| DNA-binding domains | 985 | 6615 (7372) | 2411 (2931) | 663 | 4284 (4510) | 1291 (1315) | 1.21 |
| Contacts in Domain | 607 | 3825 (4454) | 1103 (1532) | 381 | 2325 (2440) | 435 (445) | 1.54 |

*OR: Odds Ratio

**Table S7:** Description of the 31 selected features used in the final DBP-CanPred model for driver mutation prediction.

| **Feature category** | **Features** |
| --- | --- |
| **Conservation-scores** | 'VELIBIL', 'GERSTEIN','TAYLOR_NO_GAPS','SHENKIN', 'VALDAR', 'THOMPSON','NOT_LANCET' |
| **Substitution matrices** | 'AZAE970102','HENS920101','KOSJ950114','KOSJ950102',  'KOSJ950111','KOSJ950101','KOSJ950115','MIYS930101', |
| **PSSM-based features** | 'info_per_pos', 'rel_weight_to_pseudo' |
| **IDR-based features** | 'IDR_binary' |
| **Physicochemical-based features** | 'Esm', 'aN', '-TdSc','ASAD','pHi' |
| **Secondary structure** | 'psi', 'phi', 'O_NH_1_energy', |
| **Mutation-induced structural perturbations** | 'helix_charge_shift', 'loop_mutation_flex', 'proline_involved',  'saltbridge_change','aromatic_change' |

**Table S8.** Final CatBoost hyperparameter configuration used to train DBP-CanPred.

| **Parameter** | **Value** |
| --- | --- |
| Grow policy | Loss-guide |
| Iterations | 681 |
| Learning rate | 0.06907 |
| L2 leaf regularization | 17.7829 |
| Border count | 54 |
| Bagging temperature | 0.0460 |
| Random strength | 2.7589 |
| Minimum data in leaf | 13 |
| Subsample | 0.6070 |
| Column sample by level | 0.7474 |
| Maximum leaves | 32 |
| Loss function | Logloss |
| Evaluation metric | AUC |
| Early stopping rounds | 50 |

**Table S9:** Cross-Validation results for the selected best feature set.

| **Fold** | **Sensitivity** | **Specificity** | **BAC** | **F1** | **AU-ROC*** | **Coverage** |
| --- | --- | --- | --- | --- | --- | --- |
| 1 | 0.83 | 0.62 | 0.72 | 0.81 | 0.76 | 0.81 |
| 2 | 0.82 | 0.55 | 0.69 | 0.79 | 0.75 | 0.88 |
| 3 | 0.86 | 0.59 | 0.72 | 0.82 | 0.77 | 0.81 |
| 4 | 0.87 | 0.58 | 0.73 | 0.83 | 0.77 | 0.77 |
| 5 | 0.85 | 0.58 | 0.72 | 0.81 | 0.76 | 0.77 |
| Average | 0.85 | 0.58 | 0.72 | 0.81 | 0.76 | 0.81 |

*AU-ROC overall: AU-ROC including uncertain mutations	with a coverage of 100%.,

BAC: Balanced Accuracy

**Table S10:**Comparison of performance among different machine learning algorithms.

| **Dataset** | **Sensitivity** | **Specificity** | **Balanced Accuracy** | **F1** | **AU-ROC** | **Coverage** | **AU-ROC**  **Overall*** |
| --- | --- | --- | --- | --- | --- | --- | --- |
| **CatBoost** | 0.85 | 0.73 | 0.79 | 0.77 | 0.86 | 0.77 | 0.83 |
| **LightGBM** | 0.85 | 0.52 | 0.69 | 0.65 | 0.81 | 0.80 | 0.80 |
| **XGBoost** | 0.87 | 0.50 | 0.68 | 0.65 | 0.81 | 0.81 | 0.80 |

*AU-ROC overall: AU-ROC including uncertain mutations with a coverage of 100%.

**Table S11**: Feature groups used in the ablation study of DBP-CanPred.

| **Feature Category** | **Abbreviation** | **Features** |
| --- | --- | --- |
| Substitution Matrix | SM | AZAE970102, HENS920101, KOSJ950114, KOSJ950102, KOSJ950111, KOSJ950101, KOSJ950115, MIYS930101 |
| Conservation Score | CS | VELIBIL, GERSTEIN, TAYLOR_NO_GAPS, SHENKIN, VALDAR, THOMPSON, NOT_LANCET |
| Position-Specific Scoring Matrix (PSSM) | PS | rel_weight_to_pseudo, info_per_pos |
| Physicochemical Properties | PH | Esm, aN, -TdSc, ASAD, pHi |
| Intrinsically Disordered Region | IDR | IDR_binary |
| Mutation-Induced Perturbation | MP | proline_involved, saltbridge_change, aromatic_change, helix_charge_shift, loop_mutation_flex |
| Structural Constraints | SC | psi, phi, O_NH_1_energy |

**Table S12**: Performance of model training using only sequence or structure-based features on test dataset.

| **Type** | **Sensitivity** | **Specificity** | **Balanced**  **Accuracy** | **F1** | **AUROC** | **Coverage** | **AUROC**  **Overall*** |
| --- | --- | --- | --- | --- | --- | --- | --- |
| Sequence | 0.83 | 0.65 | 0.74 | 0.71 | 0.83 | 0.81 | 0.8 |
| Structure | 0.80 | 0.66 | 0.73 | 0.70 | 0.75 | 0.52 | 0.66 |

*AU-ROC overall: AU-ROC including uncertain mutations with a coverage of 100%.

| **Tool** | **Pol_U_→Pol_C_** | **Pol_U_→Pol_U_** | **H_y_→Pol_U_** | **Pol_C_→Pol_U_** | **Pol_C_→H_y_** | **H_y_→Pol_C_** | **H_y_→H_y_** | **Pol_C_→Pol_C_** | **Pol_U_→H_y_** |
| --- | --- | --- | --- | --- | --- | --- | --- | --- | --- |
| **Polyphen2** | 0.50 (0.80) | 0.59 (0.76) | 0.64 (0.82) | 0.62 (0.70) | 0.50 (0.72) | 0.69 (0.79) | 0.67 (0.75) | 0.60 (0.84) | 0.68 (0.83) |
| **MetaSVM** | 0.52 (0.80) | 0.64 (0.76) | 0.48 (0.82) | 0.54 (0.70) | 0.52 (0.72) | 0.55 (0.79) | 0.57 (0.75) | 0.53 (0.84) | 0.66 (0.83) |
| **M-CAP** | 0.41 (0.80) | 0.62 (0.76) | 0.40 (0.82) | 0.48 (0.70) | 0.49 (0.72) | 0.44 (0.80) | 0.48 (0.75) | 0.54 (0.84) | 0.50 (0.82) |
| **MutPred** | 0.58 (0.79) | 0.72 (0.77) | 0.58 (0.82) | 0.67 (0.72) | 0.58 (0.72) | 0.70 (0.76) | 0.62 (0.77) | 0.66 (0.84) | 0.68 (0.83) |
| **MVP** | 0.32 (0.78) | 0.42 (0.72) | 0.30 (0.80) | 0.30 (0.71) | 0.26 (0.72) | 0.34 (0.76) | 0.34 (0.74) | 0.29 (0.86) | 0.38 (0.82) |
| **PrimateAI** | 0.70 (0.80) | 0.80 (0.77) | 0.75 (0.82) | **0.78** (0.70) | 0.79 (0.72) | **0.81** (0.79) | **0.76** (0.75) | 0.77 (0.84) | 0.78 (0.83) |
| **DEOGEN** | 0.47 (0.80) | 0.58 (0.76) | 0.51 (0.82) | 0.47 (0.70) | 0.46 (0.72) | 0.64 (0.79) | 0.54 (0.75) | 0.50 (0.84) | 0.56 (0.83) |
| **ClinPred** | 0.60 (0.80) | 0.75 (0.76) | 0.74 (0.82) | 0.68 (0.70) | 0.58 (0.72) | 0.63 (0.79) | 0.73 (0.75) | 0.72 (0.84) | 0.72 (0.83) |
| **AlphaMissense** | 0.78 (0.82) | 0.78 (0.74) | 0.78 (0.80) | 0.78 (0.72) | **0.78** (0.71) | 0.78 (0.78) | 0.78 (0.76) | 0.78 (0.85) | 0.78 (0.84) |
| **CADD** | 0.50 (0.80) | 0.50 (0.76) | 0.50 (0.82) | 0.50 (0.70) | 0.50 (0.72) | 0.50 (0.79) | 0.50 (0.75) | 0.50 (0.84) | 0.50 (0.83) |
| **DANN** | 0.55 (0.80) | 0.56 (0.76) | 0.55 (0.82) | 0.50 (0.70) | 0.50 (0.72) | 0.50 (0.79) | 0.52 (0.75) | 0.53 (0.84) | 0.54 (0.83) |
| **PHACTboost** | 0.62 (0.80) | 0.74 (0.76) | 0.68 (0.82) | 0.75 (0.70) | 0.66 (0.72) | 0.70 (0.79) | 0.76 (0.75) | 0.80 (0.84) | 0.80 (0.83) |
| **MutFormer** | 0.38 (0.80) | 0.44 (0.76) | 0.32 (0.82) | 0.36 (0.70) | 0.34 (0.72) | 0.49 (0.79) | 0.37 (0.75) | 0.30 (0.84) | 0.40 (0.83) |
| **MutScore** | 0.54 (0.80) | 0.66 (0.76) | 0.57 (0.82) | 0.57 (0.70) | 0.62 (0.72) | 0.73 (0.79) | 0.61 (0.75) | 0.54 (0.84) | 0.60 (0.83) |
| **SIFT4G** | 0.56 (0.78) | 0.76 (0.71) | 0.62 (0.80) | 0.68 (0.70) | 0.58 (0.72) | 0.70 (0.76) | 0.70 (0.74) | 0.64 (0.86) | 0.70 (0.82) |
| **ESM1b** | 0.69 (0.80) | 0.75 (0.76) | 0.67 (0.82) | 0.72 (0.70) | 0.66 (0.72) | 0.74 (0.79) | 0.74 (0.75) | 0.80 (0.84) | 0.77 (0.83) |
| **Fathmm-XF** | 0.66 (0.80) | 0.76 (0.76) | 0.66 (0.82) | 0.71 (0.70) | 0.64 (0.72) | 0.66 (0.79) | 0.73 (0.75) | 0.76 (0.86) | 0.76 (0.83) |
| **DBP CanPred** | **0.80** | **0.76** | **0.82** | 0.70 | 0.72 | 0.79 | 0.75 | **0.84** | **0.83** |

**Table S13**: Comparison of DBP-CanPred with existing methods on different substitution categories using balanced accuracy.

*DBP-CanPred performance for corresponding dataset is shown in parentheses.

**Table S14:** DBP-CanPred predictions for CTCF protein-DNA interface mutations observed in COSMIC, ClinVar, and gnomAD.

| **COSMIC** | | | | | |
| --- | --- | --- | --- | --- | --- |
| **Samples** | **Interface Sites** | **Mutations** | **Driver** | **Neutral** | **Uncertain** |
| 1 | 27 | 40 | 23 | 5 | 12 |
| >2 | 13 | 14 | 10 | 0 | 4 |
| **ClinVar** | | | | | |
| **Clinical Significance** | **Interface Sites** | **Mutations** | **Driver** | **Neutral** | **Uncertain** |
| Pathogenic | 15 | 24 | 19 | 0 | 5 |
| Uncertain | 17 | 19 | 9 | 0 | 10 |
| **gnomAD** | | | | | |
| **Allele Frequency** | **Interface Sites** | **Mutations** | **Driver** | **Neutral** | **Uncertain** |
| 0 | 16 | 20 | 16 | 3 | 1 |
| 0 to 2.07 × 10⁻⁶ | 12 | 14 | 8 | 1 | 5 |

**Figure S1:** Comparison of local structural connectivity for driver and neutral mutations in wild-type structures

Statistical significance is indicated (*P < 0.05, **P < 0.01, ***P < 0.001).
